# Exploring the status and associated factors of the readiness for return-to-work in young and middle-aged stroke patients

**DOI:** 10.1038/s41598-024-53262-2

**Published:** 2024-02-03

**Authors:** Ziwei Liu, Jiaxin Li, Fangli Liu, Ningxiao Guan, Ye Li, Yu Zhang, Linlin Hou, Qiuhuan Jiang

**Affiliations:** 1 Nursing Department, The First Affiliated Hospital of Henan University of Traditional Chinese Medicine, No. 19 Renmin Road, Jinshui District, Zhengzhou, 450099 He Nan China; 2 Institute of Nursing and Health, Henan University, Kaifeng, 475000 He Nan China; 3 Nursing Department, Henan Provincial People’s Hospital, No.7 Weiwu Road, Jinshui District, Zhengzhou, 45003 He Nan China

**Keywords:** Health occupations, Neurology, Risk factors

## Abstract

Stroke increasingly affects individuals of working age. An accurate assessment of Readiness for Return-to-Work (RRTW) can help determine the optimal timing for RRTW and facilitate an early reintegration into society. This study investigates the current state of RRTW and the influencing factors among young and middle-aged stroke patients in China. A sample of young and middle-aged stroke patients hospitalized in a tertiary hospital in Henan Province between December 2021 and May 2022 were included in this study. A general information questionnaire and the Readiness for RRTW scale, the Social Support Rate Scale, the Stroke Self-Efficacy Scale, and the Fatigue Severity Scale were administered to the patients. Of the 203 patients successfully surveyed, 60 (29.6%) were in the pre-contemplation stage, 35 (17.2%) in the contemplation stage, 81 (39.9%) in the prepared for action-self-evaluative stage, and 27 (13.3%) in the prepared for action- behavior stage. Logistic regression analysis identified education level, monthly income, time to start rehabilitation therapy, social support, stroke self-efficacy, and fatigue severity as key factors affecting RRTW scale readiness in young and middle-aged stroke patients. The readiness of young and middle-aged stroke patients to Return-to-Work needs to be increased further. Healthcare professionals should consider the influencing factors of RRTW and design targeted intervention programs to facilitate a successful Return-to-Work and normal life.

## Introduction

Stroke is a group of acute cerebrovascular diseases characterized by cerebral blood flow interruption due to cerebral vascular rupture or obstruction, leading to loss of neurological function as the main clinical manifestation^1^. It is characterized by high morbidity, mortality, and recurrence rates^2^, and it is affecting an increasingly younger population, particularly in developing countries^3,4^. According to global statistics, about 31% of patients with stroke have an onset age of < 65 years, and patients aged under 35 years account for 9.77% of the total number of patients^5,6^. Data show that young and middle-aged patients in China account for 33% of all stroke patients and this proportion is gradually trending upwards, indicating that an increasing number of individuals of working age are affected by stroke^7^. Young and middle-aged people are the main constituents of the social labour force and are at an important stage in their careers. They are unable to Return-to-Work due to illness and disability, which has a great impact on their quality of life and self-esteem^8^. At the same time, the loss of productivity of patients is associated with a heavy economic burden on the family and wider society^9^.

Work serves as a critical means for individuals to realize their value and fulfill economic needs. It facilitates social participation, relationship building, skill development, and the discovery of life’s meaning, contributing to physical and mental health^10^. Returning to work can enhance patients’ physical function and life quality. The initial step in this process is assessing patients’ psychological readiness for returning to work^11^.

Readiness for Return-to-Work (RRTW) refers to the readiness of patients to Return-to-Work after leaving due to illness, and it is a key indicator of the level of rehabilitation and recovery after a stroke^12^. The level of the RRTW can predict the work participation status of patients after recovery, and accurate measurement of the RRTW can help patients gain an appreciation of the timing of their Return-to-Work; this can help avoid the negative impact of a late Return-to-Work on their professional role and economic recovery^13^. Existing studies on stroke patients have mainly focused on patients’ self-reported return-to-work status, and there are few reports on the level of preparation for Return-to-Work and related influencing factors. Thus, this study aimed to investigate the current situation regarding the RRTW of young and middle-aged stroke patients and analyse the influencing factors, in order to provide a reference for the development of intervention programs to promote the Return-to-Work and return to society in young and middle-aged stroke patients.

## Materials and methods

### Participants and study design

This cross-sectional descriptive correlational study was conducted between December 2021 and May 2022 in a tertiary care hospital in Henan, China. Participants were recruited through convenience sampling. The inclusion criteria were patients who: (i) Met the World Health Organization’s definition of young and middle-aged people aged 18 and 59 years^14^; (ii) Were diagnosed with stroke on imaging, non-acute patients who were admitted to the hospital for rehabilitation; (iii) Did not have mental illness or cognitive impairment; (iv) Were involved in work before the illness and have not retired; (v) Not yet successfully reintegrated into the workforce. Patients with a combination of other serious illnesses or other cerebrovascular diseases and the presence of severely impaired vision and hearing were excluded from this study.

**Figure 1:**
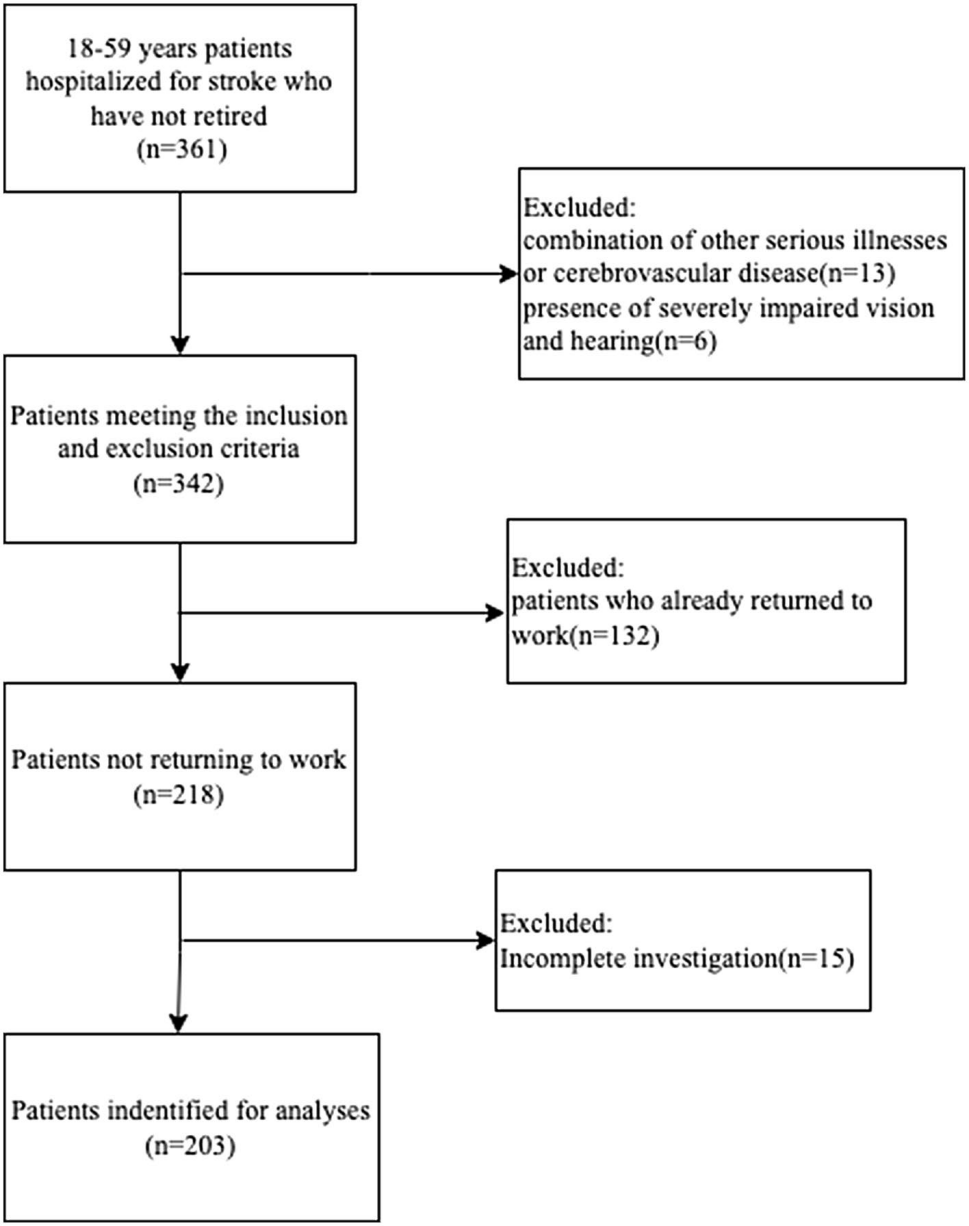
Patient screening flow chart.

According to the sample size formula for the logistic regression analysis, N = (Z_1 _− _α/2_ + Z_β_)^2^/[P_1_(1 − P_1_)]*b*^2^^15^, Z_1-α/2_ = 1.96 and Z_β_ = 1.28; furthermore, the literature review yielded P_1_ = 0.507 and b = 0.51^16^, P_1_ is the percentage or occurrence rate obtained from previous studies or literature reviews, b is the size of the effect size. which when incorporated into the formula yielded: N = 162. In total, 203 young and middle-aged stroke patients were included in this study. The specific screening process is shown in Fig. 1.

### Research instruments

#### Self-generated general information questionnaire

This scale was designed by the researcher and covered socio-demographic information such as age, gender, place of residence, housing status, education level, marital status, monthly income, and mode of payment for medical care. Disease-related information included the type of stroke, whether it was the first-onset stroke, the years since the stroke, at what point rehabilitation therapy was started, and whether any residual functional impairment existed. Work-related information collected included the type of occupation and nature of work before illness. In China, the types of medical insurance mainly include urban employee medical insurance and new rural cooperative medical insurance. Therefore, in the study, medical payment methods are divided into medical insurance (urban employee medical insurance and new rural medical insurance) and self-payment.

#### Readiness for return-to-work scale (RRTW)

Developed by Franche et al.^17^ and translated and revised by Cao^18^, the RRTW is mainly used to evaluate the level of a patient’s readiness for return-to-work. The scale includes 6 dimensions and 22 items and is divided into two parts. The first part comprises 4 dimensions (13 items in total) for patients who have not returned to work, including a pre-contemplation dimension, contemplation dimension, and the dimension of prepared for action-self-evaluative and prepared for action-behavioral. In the second part, there are two dimensions (9 items in total) for patients who have returned to work, namely, uncertain maintenance and active maintenance. Each item is scored on a five-point Likert scale. The total score is not set in the scale, and the sum of items in each dimension is the score of this dimension. The dimension with the highest score represents the preparation stage of the patient; the higher the stage, the higher the level of RRTW, and the more fully prepared they are to Return-to-Work. If the final score of the two dimensions is the same, the respondent will be classified as the stage at a low level in the two dimensions according to the patient’s filling results. If three or more dimensions with the same score are obtained simultaneously, the questionnaire will be deemed invalid. This study targeted patients who did not Return-to-Work by using only the first part of the scale. The scale’s internal consistency Cronbach’s α coefficient of the questionnaire in the formal investigation of this study was 0.728.

#### Social support rate scale (SSRS)

Formulated by Xiao^19^ in 1994, the SSRS includes 10 items spread across three dimensions: objective support (items 2, 6, and 7), subjective support (items 1, 3, 4, and 5), and utilization of social support (items 8, 9, and 10). The total score ranged from 22 to 66, with higher scores indicating higher levels of social support. Since its establishment, the SSRS has been widely used in 20 disciplines and majors in China, the internal consistency Cronbach’s α coefficient for this scale was 0.941.

#### Stroke self-efficacy questionnaire (SSEQ)

Developed by Jones et al.^20^, Li et al.^21^ translated the SSEQ into Chinese and applied it to patients with first-episode stroke to measure functional performance and self-management confidence levels during the recovery period of stroke. Internal consistency Cronbach’s α coefficient of the scale was 0.969. The scale has 13 items in total, divided into two dimensions: activity function (8 items) and self-management (5 items). A Likert 10-level scoring method is used. The higher the total score, The higher the rehabilitation self-efficacy of the subjects. The Cronbach’s α coefficient of the questionnaire in the formal investigation of this study was 0.947.

#### Fatigue severity scale (FSS)

Compiled by Krupp et al.^22^ in 1989, Wu and Wang^23^ translated the FSS into Chinese and applied it to evaluate the fatigue severity of stroke patients. The scale consists of a single dimension comprising nine items, all of which are scored using a 7-level Likert scale. The total score is either the average or sum of the nine items. According to the Chinese version of the scoring criteria, a total FSS score of ≥ 36 or an average score of ≥ 4 indicates fatigue^24^, with higher scores indicating a more severe level of fatigue. The reliability and validity of the Chinese version were good, with an internal consistency Cronbach’s α coefficient of 0.932 and an intra-group correlation coefficient of 0.742^24^, This indicates that the FSS is a reliable and effective tool for measuring post-stroke fatigue. In this study, the Cronbach’s α coefficient of the questionnaire was 0.938.

### Data collection procedure

Regarding the pre-designed questionnaire, Questionnaire Star software was used to fill in the questionnaire online or distribute the questionnaire in person to collect data. The purpose and benefits of the study were explained to the patients before filling in the questionnaire, and the patients were asked to complete the questionnaire themselves as much as possible. If the patient’s education level, physical function, and other aspects could not be filled in by the patient, the patient’s family members or researchers were allowed to assist them in completing the questionnaire. All questionnaires were collected and checked by researchers, and those with incomplete information were excluded. A total of 218 questionnaires were distributed and 203 were recovered, with an effective recovery rate of 93.1%.

### Statistical analysis

SPSS 26.0 software (IBM Corp, Armonk, NY, USA) was used for the statistical analysis and processing of all data. Means ± standard deviations were used to describe the measures, and counts were expressed as the number of cases and rates (%); Univariable analysis were conducted using rank sum tests such as the Mann–Whitney U test or the Kruskal–Wallis H test: Spearman’s correlation analysis was used to explore the relationship between RRTS and self-efficacy, social support, fatigue severity scale, and the respective dimensions of each scale. Ordered multiple logistic regression was used for multifactor analysis, with *P* < 0.05 being considered a statistically significant difference.

### Ethical approval and consent to participate

The study was approved by the Ethics Committee of the College of Nursing and Health of Henan University (HUSOM2021-288), and all methods were carried out according to relevant guidelines and regulations. All patients in the study gave informed consent before the questionnaire began.

## Results

### Sociodemographic characteristics of the participants

Of the 203 patients surveyed, more than half were male (60.1%), most were aged 50–59 years (42.4%), and 29.6% had a secondary school level of education. In terms of monthly income, 39.9% of the patients had a monthly income of RMB 3,000–4,999. The majority of patients had an ischaemic-type stroke, 54.7% had experienced their first stroke, and only 7.4% had a history of stroke longer than 3 years. In addition, more than half of the patients (n = 128, 63.1%) had the occupation type of knowledge worker. The socio-demographic characteristics of the participants are shown in Table 1. Note: Functional impairment: The Barthel index scale was used to assess whether the patient had dysfunction. patients with a score of 60 or less were defined as having dysfunction. The absence of functional impairment therefore does not mean complete recovery from stroke see.

**Table 1:** Sociodemographic characteristics of the participants (n = 203).

Variables		n (%)
Gender	Male	122 (60.1%)
	Female	81 (39.9%)
Age (years)	18–29	16 (7.9%)
	30–39	45 (22.2%)
	40–49	56 (27.6%)
	50–59	86 (42.4%)
Place of residence	Town	111 (54.7%)
	Rural	92 (45.3%)
Living alone	Yes	36 (17.7%)
	No	167 (82.3%)
Educational level	Primary and below	11 (5.4%)
	Middle school	60 (29.6%)
	High school	35 (17.2%)
	Associate College	41 (20.2%)
	Bachelor and above	56 (27.6%)
Marital status	Married	162 (79.8%)
	Unmarried	24 (11.8%)
	Other situations	17 (8.4%)
Monthly income (RMB)	< 1000	10 (4.9%)
	1000–2999	66 (32.5%)
	3000–4999	81 (39.9%)
	> 5000	46 (22.7%)
Type of medical payments	Town/Employee medical insurance	91 (44.8%)
	New rural cooperative insurance	102 (50.2%)
	Self-financing	10 (4.9%)
Stroke type	Ischaemic stroke	94 (46.3%)
	Intracerebral hemorrhage	68 (33.5%)
	Mixed type	41 (20.2%)
First attack	Yes	111 (54.7%)
	No	92 (45.3%)
Years since stroke	≤ 1 year	158 (77.8%)
	1–3 years	30 (14.8%)
	> 3 years	15 (7.4%)
Time to start rehabilitation therapy	< 7 days	92 (45.3%)
	7–15 days	56 (27.6%)
	> 15 days	55 (27.1%)
Functional impairment	Yes	75 (36.9%)
	No	128 (63.1%)
Nature of occupation	Knowledge worker	128 (63.1%)
	Manual worker	75 (36.9%)
Occupation type	Farmer	55 (27.1%)
	Worker	72 (35.5%)
	Self-employed person	52 (25.6%)
	Administrative, Corporate, Institutional units	17 (8.4%)
	Other	7 (3.4%)

### Distribution of the dimensions of readiness for return-to-work among young and middle-aged stroke patients

The results showed that 60 patients (29.6%) in this group were in the pre-contemplation stage, 35 (17.2%) in the contemplation stage, 81 (39.9%) in the prepared for action-self-evaluative stage, and 27 (13.3%) in the prepared for action-behavioral.

### Demographic univariable analysis of influences on readiness for return-to-work in young and middle-aged stroke patients

The results of the univariable analysis showed that place of residence, education level, monthly income, mode of payment of medical expenses, Years since stroke, time of starting rehabilitation therapy, type of occupation, and nature of occupation had an impact on the RRTW of young and middle-aged stroke patients, with statistically significant differences (*p* < 0.05), as shown in Table 2.

**Table 2:** Univariable analysis.

Variables		P	I	E	B	H or Z	*P*
Gender	Male	32	23	45	22	− 1.607	0.108
	Female	28	12	36	5		
Age (years)	18–29	8	1	7	0	3.197	0.362
	30–39	12	5	24	4		
	40–49	12	17	16	11		
	50–59	28	12	34	12		
Place of residence	Town	41	14	47	9	− 2.216	0.027
	Rural	19	21	34	18		
Living alone	Yes	6	5	21	4	− 1.702	0.089
	No	54	30	60	23		
Educational level	Primary and below	5	5	0	0	35.589	< 0.01
	Middle school	8	16	22	14		
	High school	6	4	21	4		
	Associate College	9	4	20	8		
	Bachelor and above	32	5	18	1		
Marital status	Married	50	28	60	24	0.399	0.819
	Unmarried	7	3	13	1		
	Other situations	3	4	8	2		
Monthly income (RMB)	< 1000	5	0	5	0	14.688	0.002
	1000–2999	13	19	23	11		
	3000–4999	17	11	41	12		
	> 5000	25	5	12	4		
Type of medical payments	Town/Employee medical Insurance	41	9	33	8	12.061	0.002
	New rural cooperative insurance	19	24	41	18		
	Self-financing	0	2	7	1		
Stroke type	Ischaemic stroke	34	17	32	11	3.917	0.141
	Intracerebral haemorrhage	17	11	28	12		
	Mixed type	9	7	21	4		
First attack	Yes	35	14	45	17	− 0.474	0.636
	No	25	21	36	10		
Years since stroke	≤ 1 year	46	29	64	19	8.397	0.015
	1–3 years	14	3	9	4		
	> 3 years	0	3	8	4		
Time to start rehabilitation therapy	< 7 days	45	8	36	3	23.602	< 0.01
	7–15 days	5	17	22	12		
	> 15 days	10	10	23	12		
Functional impairment	Yes	23	13	29	10	− 2.360	0.814
	No	37	22	52	17		
Nature of occupation	Knowledge worker	34	8	69	17	− 3.175	0.001
	Manual worker	26	27	12	10		
Occupation type	Farmer	20	17	14	4	3.835	0.280
	Worker	32	20	13	7		
	Self-employed person	16	17	13	6		
	Administrative, Corporate, Institutional units	5	2	6	4		
	Other	2	1	3	1		

P = Pre-contemplation stage; I = Contemplation stage; E = Prepared for action-self-evaluative stage; B = Prepared for action-behavioral stage; H = Kruskal–Wallis H test; Z = Mann–Whitney U test.

### Correlation between readiness for return-to-work and social support, stroke self-efficacy, and fatigue severity in young and middle-aged stroke patients

The results of the study showed that RRTW among young and middle-aged stroke patients was positively correlated with social support and all dimensions, Stroke Self-Efficacy in stroke rehabilitation was positively correlated with activity function and self-management. and negatively correlated with the severity of fatigue, all with statistically significant differences (*p* < 0.01; see Table 3 for details).

**Table 3:** Relevance analysis.

Variables	Readiness for return-to-work (r)	*P*
Social support	0.837	< 0.001
Objective support dimension	0.672	< 0.001
Subjective support dimension	0.742	< 0.001
Support utilization dimension	0.742	< 0.001
Recovery self-efficacy	0.760	< 0.001
Activity function dimension	0.706	< 0.001
Self-management dimension	0.657	< 0.001
Fatigue severity	− 0.554	< 0.001

### Multivariable analysis of readiness for return-to-work in young and middle-aged people with stroke

The stage of RRTW in young and middle-aged stroke patients was used as the dependent variable (pre-contemplation stage = 1, contemplation stage = 2, prepared for action-self-evaluative stage = 3, prepared for action- behavioral stage = 4, with prepared for action- behavioral stage as the reference), To eliminate the interference of other meaningless variables, the statistically significant individual variables in the univariable analysis and social support, stroke self-efficacy and fatigue severity as independent variables were used in a multivariable logistic regression analysis. To facilitate data analysis, all categorical variables within these variables were recoded (Table 4). The type of health insurance was transformed into a dummy variable as it was a disorderly multi-classification variable. In this study, we used the P-value, 0.05 as criteria for parallel line test and the parallel line assumption turned out to be satisfied, which is prerequisite for ordered logistic regression analysis^25^. The results of the Multivariable analysis showed that education level, monthly income, time to start rehabilitation therapy, social support, stroke self-efficacy, and severity of fatigue were the main factors affecting the RRTW of young and middle-aged stroke patients (Table 5).

**Table 4:** Description of the assignment.

Index	Description of the assignment
Place of residence (X_1_)	0 = Town; 1 = Rural
Educational level (X_2_)	1 = Primary and below; 2 = Middle school; 3 = High school; 4 = Associate College; 5 = Bachelor and above
Monthly income (X_3_)	1 = < 1000; 2 = 1000–2999; 3 = 3000–4999; 4 = ≥ 5000
Type of medical payments (X_4_) (Town/employee medical insurance)	(1, 0) = New rural cooperative insurance; (0, 1) = Self-financing
Years since stroke (X_5_)	1 = ≤ 1; 2 = 1–3; 3 = > 3
Time to start rehabilitation therapy (X_6_)	1 = < 7/d; 2 = 7–15/d; 3 = > 15/d
Occupation type (X_7_)	0 = Manual worker; 1 = Knowledge worker
Social support (X_8_)	Raw value input
Stroke self-efficacy (X_9_)	Raw value input
Fatigue severity (X_10_)	Raw value input
Readiness for return-to-work (Y)	1 = Pre-contemplation; 2 = Contemplation; 3 = Prepared for action-self-evaluative; 4 = Prepared for action-behavioral

**Table 5:** Multivariable analysis.

Variables	*B*	*S.E*	*Wald*	*P*	*OR*	95% CI	
						Low	Top
Educational level	1.564	0.660	5.618	0.018	4.778	1.311	17.409
Monthly income	1.349	0.645	4.373	0.037	3.854	1.089	13.640
Time to start rehabilitation therapy	− 1.387	0.659	4.43	0.035	0.250	0.069	0.909
Social support	0.285	0.037	59.310	< 0.001	1.330	1.236	1.430
Stroke self-efficacy	0.050	0.013	15.590	< 0.001	1.051	1.025	1.078
Fatigue severity	− 0.111	0.036	9.510	0.002	0.895	0.834	0.961

## Discussion

The results of this study showed that there were 95 patients in the pre-contemplation and contemplation stage, accounting for 46.8% of the total number of patients, which is similar to the research results reported by researchers in other parts of China (52.9%^26^ and 47.06%^27^). In previous studies, Chen et al.^27^ focused on exploring the factors influencing patients who had successfully returned to work and did not focus on the needs of patients who had not yet returned to work, Another study focused on the effects of stigma, knowledge of the disease, and mobility on patients’ RRTW^26^. In contrast, this study focused on the needs of patients who had not yet returned to work and, for the first time, included the effects of stroke self-efficacy, fatigue severity, stroke type, and other variables on the RRTW process, making the inclusion of factors more comprehensive.

The results of this survey suggest that that most patients did not have the intention of returning to work at the time of the survey. The reason may be that these patients may lack the information, medical support, self-efficacy, and other social support needed to Return-to-Work. Compared with foreign research^28^, the number of people prepared for action- behavioral in this study is relatively small, which is due to the fact that the intervention on returning to work was carried out earlier in foreign countries, and they have a complete vocational rehabilitation training system and professional vocational rehabilitation doctors. This suggests that in the future, China’s stroke rehabilitation should learn from the experience of foreign vocational rehabilitation and train vocational rehabilitation doctors and nurses who can conduct vocational rehabilitation assessments for patients after treatment, formulate vocational rehabilitation plans, help with the transition to vocational rehabilitation, and provide information on employment guidance. To promote the change of patients’ Return-to-Work behavior and conduct follow-up work. In addition, examples of successful Return-to-Work should be described to patients; through this, the power of example is used to strengthen self-worth and improve self-efficacy and confidence in returning to work, to help patients Return-to-Work smoothly.

The survey shows that education level is the main influencing factor in the readiness of young and middle-aged stroke patients to Return-to-Work. This may be because well-educated people have higher expectations for disease rehabilitation, are more able to express their needs for medical care and subsequent rehabilitation, and have better compliance behavior^29^. In addition, patients with higher education levels are more likely to engage in mental work and jobs with higher pay, thus having stable job security^30^. However, patients with low education are mainly engaged in manual labour, and their income is not fixed. Therefore, they cannot Return-to-Work smoothly after a stroke. Patients with a higher monthly income (RMB > 5000) have higher RRTW, which is consistent with previous research results^29^ and is possible because those with a higher income have higher socioeconomic status, greater job security, and better welfare security as well as employment terms and conditions. Moreover, people with higher incomes have a better economic foundation can afford expensive medical expenses, and enjoy more elaborate rehabilitation services. Patients who started rehabilitation therapy earlier (< 7 days) had higher levels of RRTW. The reason may be that early rehabilitation is a critical period for promoting the plasticity of neural function, and early rehabilitation therapy is conducive to improving the cerebral blood supply of patients and the compensatory repair of tissues around lesions, thus promoting the recovery of the limb motor function of patients^31^. A retrospective investigation showed that the professional support and participation of occupational physicians in the early stages of stroke were positive prognostic factors for patients to Return-to-Work^32^, indicating that the timing and timeliness of rehabilitation therapy are also important factors to ensure that patients can Return-to-Work earlier. Therefore, medical staff should pay particular attention to the needs of stroke patients with low income and low education levels to facilitate their Return-to-Work and provide them with guidance in terms of re-employment, medical security, and welfare policies in an easy-to-understand way. In addition, when formulating intervention measures, it is necessary to intervene in the rehabilitation therapy of stroke patients as early as possible. Seizing the opportunity during the optimal time window will facilitate the recovery of physical function.

The results of this study indicate that the higher the amount of social support, the higher the stage of a patient’s RRTW. Young and middle-aged stroke patients of working age mainly need support from their families, work units, and medical institutions^33^. Emotional support from relatives can avoid the breeding of patients’ negative emotions, reduce the internal emotional damage caused by the disease, promote the evolution of the disease in a good direction, and then improve the level of Return-to-Work^34^. For middle-aged and young stroke patients of working age, patients need not only care and help from their families, but also support from work, medical institutions, and the government. Nicholas et al.^35^ found that early contact and communication between work units and patients, help to coordinate posts, and provision of accommodation could enable patients to readjust to work. Professional vocational rehabilitation (job assessment, job analysis, job placement, etc.) can also help to address the work readiness needs of stroke patients. In a Dutch study, after 4 months of vocational rehabilitation, 86% of patients successfully returned to work, with an average working time increased by 5.3 h compared to before the intervention, and 64% of patients were still on the job 3 to 6 years later^36^. Given that social support is a controllable influencing factor, clinical medical staff should pay attention to mobilizing a patient’s social support system, strengthen the interaction and cooperation between the medical system, community, work unit, and patient’s family, and help patients to achieve their work value to the maximum extent through multi-disciplinary cooperation and efforts.

Our results showed that the level of a patient’s self-efficacy was positively correlated with the readiness of the patient to Return-to-Work; that is, the higher the level of self-efficacy, the stronger the willingness of the patient to Return-to-Work, and the higher the readiness of the patient to Return-to-Work. The overall score (77.20 ± 22.53) among the 203 patients in this study was at a low level, which was consistent with the investigation results of Liang et al.^37^ This indicates that patients generally lack confidence in their rehabilitation and do not perceive the benefits of returning to work and the seriousness of non-compliance. This may be due to the fact that most stroke patients in this study, whose onset time is less than 1 year, are in the initial stage of rehabilitation and feel useless when they suddenly leave the work state. Meanwhile, high medical costs have caused certain economic burden and heavy psychological pressure on patients, thus affecting their confidence in rehabilitation. So, it is difficult to keep a positive attitude towards one’s own condition after illness, and it is easier to escape from the difficulties and obstacles encountered in the preparation process of returning to work, leading to further obstruction to returning to work. Good self-efficacy can improve a patient’s exercise compliance, self-management ability, daily activity ability, and memory level, and then improve the rehabilitation outcome^37^. Studies have shown that encouragement from others, the experience of patients who have returned to work, and the sharing of social welfare policies can all help to promote a change in a patient’s current state, improve a patient’s positive beliefs, and arouse the determination of positive behaviour change^38^. Therefore, when formulating intervention measures, it is necessary to pay attention to the psychological intervention of patients and correct erroneous disease cognition. Nurses should carry out targeted health education for patients at all stages of rehabilitation treatment, enhance disease knowledge, let patients face their disease and future lives with dignity and optimism, and help improve their confidence about returning to work.

Post-stroke fatigue is a common and long-term complication after stroke, and its prevalence ranges from 25 to 85%^39^. The results of this study showed that 59.6% of patients had post-stroke fatigue, The more fatigued the patient, the lower the level of RRTW. Previous studies have shown that patients with post-stroke fatigue have a lower chance of returning to paid work and have a significantly reduced workload after returning to work^40,41^. On the one hand, after fatigue symptoms occur, patients will continue to feel inadequate physical strength, burnout, or difficulty in maintaining daily activities, thus hindering the rehabilitation process of patients^42^. On the other hand, fatigue can cause anxiety, depression, anger, and other negative emotions, further reducing the ability to Return-to-Work, prolong the Return-to-Work time, or reduce the return-to-work rate^43^. In addition, a survey found that 30% of patients’ relatives regarded fatigue after stroke as a manifestation of patients’ laziness, which led to family conflicts and aggravated patients’ negative emotions^44^. This indicates that the influence of fatigue on patients after stroke is not only limited to the ability to work, but it also affects the mental health of patients and hinders the process of returning to society and work. Therefore, medical staff should add strategies to cope with post-stroke fatigue into the content about returning to work, carrying out fatigue management for patients after stroke in the recovery period, and giving interventions such as mindfulness-based stress reduction, sleep management, and exercise to improve fatigue, so as to improve the level of patients’ RRTW.

It is worth mentioning that, instead of treating the results of the scale in a hierarchical manner, the present study recoded the other categorical variables and entered them into the regression equations along with the continuous variables of the scale, which allowed for a more sensitive observation of the effects of changes in levels of social support, stroke self-efficacy, and fatigue severity on RRTW.

Our study has some limitations. (i) The sample was only recruited from a single tertiary hospital in Zhengzhou, China. Therefore, the convenience sampling method was adopted and may not be representative of the various ethnic and racial populations in China. (ii) The time after stroke has a great impact on patients’ RRTW, especially in the first year. However, this study did not divide the time period of stroke in more detail, which may cause some bias. (iii) we used a cross-sectional study design that could not establish a causal relationship between potential risk factors and RRTW. In the future, researchers can strengthen the study design by increasing the sample size, expanding the scope of the investigation, and combining qualitative and quantitative methods to further explore the relationship between the readiness of young and middle-aged stroke patients to Return-to-Work and other factors.

## Conclusion

The return-to-work level of young and middle-aged stroke patients needs to be further improved and is affected by the patient’s self-efficacy, social support, degree of fatigue, education level, monthly income, and the time taken to start rehabilitation therapy. It is suggested that primary healthcare institutions and rehabilitation service institutions should pay more attention to young and middle-aged stroke patients, improve the assessment, and discover the needs of patients in a timely manner during the process of returning to work. It is important to fully mobilize patients’ families, colleagues, employers, society, and medical and healthcare personnel from both a holistic and systematic perspective, in order to construct a multi-dimensional intervention strategy, so as to achieve the goal of a healthy and high-quality Return-to-Work for stroke patients.

## Data Availability

The data that support the findings of this study are available from the corresponding author upon reasonable request.
